# Hybrid-Mechanism Distributed Sensing Using Forward Transmission and Optical Frequency-Domain Reflectometry

**DOI:** 10.3390/s25196229

**Published:** 2025-10-08

**Authors:** Shangwei Dai, Huajian Zhong, Xing Rao, Jun Liu, Cailing Fu, Yiping Wang, George Y. Chen

**Affiliations:** 1 State Key Laboratory of Radio Frequency Heterogeneous Integration, Key Laboratory of Optoelectronic Devices and Systems of Ministry of Education/Guangdong Province, College of Physics and Optoelectronic Engineering, Shenzhen University, Shenzhen 518060, China; 2350453003@email.szu.edu.cn (S.D.); 2250453001@email.szu.edu.cn (H.Z.); raoxing@szu.edu.cn (X.R.); fucailing@szu.edu.cn (C.F.); ypwang@szu.edu.cn (Y.W.); 2 Shenzhen Key Laboratory of Ultrafast Laser Micro/Nano Manufacturing, Guangdong and Hong Kong Joint Research Centre for Optical Fibre Sensors, Shenzhen University, Shenzhen 518060, China; 3 Institute of Microscale Optoelectronics, Shenzhen University, Shenzhen 518060, China; liu-jun-1987@live.cn

**Keywords:** fiber optic sensing, hybrid system, forward transmission, low-frequency vibration, OFDR

## Abstract

**Highlights:**

**What are the main findings?**

- This is the first study to combine forward transmission distributed sensing and optical frequency-domain reflectometry.
- A balanced system can be established to provide a middle point between the advantages of the two technologies.

**What is the implication of the main finding?**

- The hybrid system bridges a critical gap in distributed fiber-optic sensing and can offer new insights into possible hybrid systems, making it suitable for the combined measurement of dynamic (e.g., gas leakage, pipeline excavation warning) and quasi-static (e.g., pipeline displacement) events in long-distance applications.

**Abstract:**

Fiber-optic sensing systems based on a forward transmission interferometric structure can achieve high sensitivity and a wide frequency response over long distances. However, there are still shortcomings in its ability to position multi-point vibrations and detect low-frequency vibrations, which limits its usefulness. To address these challenges, we study the viability of merging long-range forward-transmission distributed vibration sensing (FTDVS) with high spatial resolution optical frequency-domain reflectometry (OFDR), forming the first reported hybrid distributed sensing method between these two methods. The probe light source is shared between the two sub-systems, which utilizes stable linear optical frequency sweeping facilitated by high-order sideband injection locking. As a result, this is a new approach for the FTDVS method, which conventionally uses fixed-frequency continuous light. The method of nearest neighbor signal replacement (NSR) is proposed to address the issue of discontinuity in phase demodulation under periodic external modulation. The experimental results demonstrate that the hybrid system can determine the position of vibration signals between 0 and 900 Hz within a sensing distance of 21 km. When the sensing distance is extended to 71 km, the FTDVS module can still function adequately for high-frequency vibration signals. This hybrid architecture offers a fresh approach to simultaneously achieving long-distance sensing and wide frequency response, making it suitable for the combined measurement of dynamic (e.g., gas leakage, pipeline excavation warning) and quasi-static (e.g., pipeline displacement) events in long-distance applications.

## 1. Introduction

Distributed optical fiber vibration sensors (DOFVSs) have attracted considerable research interest in the past few decades because of the unique advantages of optical fiber, such as long-distance signal delivery, high sensitivity, intrinsic inertness, resilience to electromagnetic interference, etc. [1,2]. DOFVS technology can be divided into two main categories. One type is dependent on Rayleigh backscattering (RBS), such as phase-sensitive optical time-domain reflectometry (φ-OTDR), which offers a relatively long sensing distance and high sensitivity [3,4]. The other type is OFDR, which possesses high spatial resolution (SR) and the ability to undertake static strain measurements in addition to dynamic strain (vibration) measurements [5,6]. However, RBS signals are several orders of magnitude weaker than forward propagating signals; thus, they limit the sensing distance. In order to meet the ultra-long sensing distance requirements of certain applications, including ocean activities monitoring [7], earthquake early-warning systems [8], and smart railways [9], another type of DOFVS known as forward transmission distributed vibration sensing (FTDVS) is emerging as a viable alternative to φ-OTDR and OFDR technologies. These sensing systems utilize the interference between light propagating in the forward direction instead of the backscattering light, resulting in a longer sensing distance, higher sensitivity, wider frequency response and lower computational load. This approach facilitates a simple pathway to realizing sensing distances of up to hundreds of kilometers [10,11,12,13]. Hence, it is highly applicable in large-scale intrusion detection [14], structural monitoring [15,16], and perimeter security [17]. Q. Chen et al. introduced a FTDVS system based on a dual Mach–Zehnder interferometer (DMZI), achieving a positioning accuracy of ±50 m with a multi-span sensing range of 320 km when disturbances are above 1 MHz [18]. Yaxi Yan et al. introduced a FTDVS using forward transmission, coherent detection, and a frequency-shifted optical delay line to achieve a multi-span sensing range of 1230 km [19]. G. Marra et al. successfully detected earthquakes over terrestrial and submarine links with fiber cable lengths ranging from 75 to 535 km [20]. Kong W et al. proposed an FTDVS system based on phase difference and end-point amplification, allowing for a positioning accuracy within ±51 m between 300 Hz and 10 kHz over a 122 km sensing range [21].

However, FTDVS is still an emerging technology that face challenges in resolving the position of many vibration events occurring simultaneously, as well as detecting static or low-frequency vibration signals. Researchers are striving to effectively address the positioning of complex multi-point vibration events. Ye et al. employed the cepstrum method to achieve multi-point positioning, but the signal processing complexity would become an issue if anything other than two vibration signals were present [22]. Teng et al. utilized the spectral peak ratio of vibration signals in the frequency domain to determine positions. However, when the amplitudes of multiple vibrations are similar, the positioning accuracy is reduced [23]. B. Ma et al. introduced a distributed fiber-optic vibration sensing employing position algorithm based on phase-spectrum time delay estimation, and tested a 40 km length optical fiber cable. However, these methods all suffer from strong low-frequency noise and the inability to perform static strain measurements [24]. In addition, some studies have used a hybrid FTDVS/φ-OTDR integration in an attempt to solve this problem. P. Ma et al. proposed an integrated vibration measurement scheme with a wide dynamic range, high frequency response and multi-point vibration positioning by combining a unidirectional Mach–Zehnder interferometer (MZI) and φ-OTDR [25]. Q. He et al. proposed hybrid system of MZI and φ-OTDR which used two types of pulse light with different widths as probe light [26]. Although these methods overcame the problem of high-frequency response in DOFVS systems, RBS is relied upon for vibration positioning, which means the maximum sensing distance is bottlenecked. Additionally, since they use laser sources with different wavelengths/frequencies, crosstalk from the other source may still be present at the receiver. Liu et al. proposed a time-gated digital optical frequency-domain reflectometry (TGD-OFDR) technique using chirped swept pulse light, which resulted in a high spatial resolution (1.6 m) over a long sensing distance (110 km) [27]. However, TGD-OFDR is still based on Rayleigh backscattering, and the attenuation of weak backscattered light after long-distance transmission still limits its sensing distance.

To address the simultaneous requirement of long-sensing distance, multi-point vibration positioning and low-frequency signal detection, we propose a hybrid distributed sensing technology based on a first-reported combination of FTDVS and OFDR. The shared probe light utilizes stable linear optical frequency sweep facilitated by high-order sideband injection locking, which is a new approach for FTDVS that otherwise uses fixed frequency. The method of nearest neighbor signal replacement (NSR) is proposed to address the issue of discontinuity in phase demodulation under periodic external modulation, which can effectively reduce the impact of dead-zone detection on positioning results. The proposed hybrid system serves as a proof-of-concept study for integrating distinct sensing optical signals from two different sensing methods. As such, the hybrid system provides an all-round performance across the board, instead of making a breakthrough in any particular area. By virtue of their inherent data redundancy, hybrid sensing systems enable multi-dimensional information fusion, thereby enhancing reliability and robustness in complex sensing scenarios.

## 2. Experiment Design

**Figure 1:**
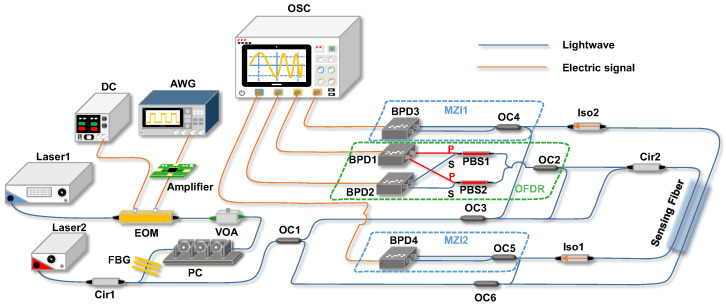
Schematic of the hybrid sensing system. AWG: arbitrary waveform generator; EOM: electro-optic modulator; VOA: variable optical attenuator; PC: polarization controller; FBG: fiber Bragg grating; OC: optical coupler; Cir: circulator; SMF: single-mode fiber; PZT: piezoelectric ceramic transducer; Iso: isolator; BPD: balanced photodetector; PBS: polarization beam splitter; OFDR: optical frequency-domain reflectometer; MZI: Mach–Zehnder interferometer; FTDVS: forward-transmission distributed vibration sensor.

Both OFDR and FTDVS aspects in the hybrid distributed fiber-optic sensing system use linearly swept wavelength for the probe light, and the two modules can achieve synchronous data acquisition. While internal laser source modulation can achieve a broader sweep range, this method suffers from sweep nonlinearity and can degrade the linewidth of the narrow-linewidth laser source. To mitigate these issues, the proposed system employs a high-order sideband injection-locking technique. This method utilizes an electro-optic modulator (EOM) to modulate a single-frequency narrow-linewidth laser source. The linearity of the frequency sweep is determined by the swept electrical signal generated by a high-precision arbitrary wavelength generator (AWG). To prevent sideband aliasing caused by wide-bandwidth modulation, optical injection locking locks the modulated light to the fourth-order sideband. This approach enhances the single-sideband suppression ratio while simultaneously enables a broader sweep range. [28]. Figure 1 shows the experiment setup of the hybrid distributed sensing system. Laser1 (Koheras BASIK E15, NKT Photonics) generates continuous-wave light with a center frequency of 1549.7 nm and 1 kHz linewidth. After electro-optic modulation (EOM), it produces multi-order sidebands, which are injected into Laser2 through the circulator (Cir1). The drive signal of the EOM comes from the amplified and chirped sweep signal output by an arbitrary waveform generator (AWG). The variable optical attenuator (VOA) and polarization controller (PC) are used to adjust the power and state of polarization of the optical frequency sweep, respectively. The fiber Bragg grating (FBG) is employed to filter out the carrier of the high-order sideband spectrum before injecting to a tunable laser denoted by Laser2. By adjusting the drive voltage of Laser2, the center frequency of the output light of Laser2 can be adjusted to the fourth sideband of Laser1, which can generate optical frequency sweep with a range of 1 GHz. The linear optical frequency sweep enters the sensing fiber through the Cir2, with demodulation ends comprising OFDR, MZI1, and MZI2 (the latter two belong to forward transmission). To avoid crosstalk between the forward-propagating light and the backscattered light, two parallel coils of single-mode fiber (SMF) are deployed as the sensing link. As a future study, optical frequency shifting can be considered to shift either the Rayleigh backscatter beat signal or the forward-propagating light to a different frequency. At the receiver end, the forward-propagating signal and the Rayleigh backscattered signal can be extracted separately through frequency-domain separation. This approach can theoretically enable both modules to share the same single sensing fiber. The total length of the sensing fiber is 21,127 m. The piezoelectric ceramic transducer (PZT) was placed at a position of 20,096 m on the sensing fiber link as the vibration source. Two sections of 15 m fibers were wrapped around the same PZT to allow vibrational strain to transfer to both optical fibers.

In this system, the interference paths of the OFDR are as follows:

(1a) Cir2 → sensing fiber→ Iso1.

(1b) OC3 → OC2.

The probe light of the OFDR is divided into two paths via the optical coupler (OC3). One path enters (1b) as the reference signal, while the other path enters (1a) as the sensing signal. The RBS light from the sensing fiber is mixed with the reference signal in OC2, and the interference signal is sent to two PBSs and then detected by a pair of balanced photodetectors BPD1, BPD2 (Thorlabs PDB470C-AC, 400 MHz bandwidth). The isolator (Iso1) is used to filter any backscattering of interference light from OC5.

The sensing light of MZI1 is output from Cir2 and interferes with the reference light output from OC6. Then the interference signal is detected by BPD4. The beat frequency at the BPD4 is approximately 10 MHz, the beat frequency at the BPD1 is approximately 20 MHz, and the sampling rate of the oscilloscope (Picoscope 6426E, bandwidth = 1 GHz) is set to 78 MS/s. The interference paths of MZI1 are as follows:

(2a) Cir2 → sensing fiber → Iso1 → OC5

(2b) OC6 → OC5

Assuming the sweep rate of AWG is γ, the reference signal of MZI denoted by $E_{r,d}$ can be expressed as

(1) $$E_{r,d}(t)=E_{0}\exp\{j[2\pi f_{0}t+\pi \gamma t^{2}+\phi (t)]\},d=1,2$$

where $f_{0}$ is the initial frequency of the laser source, ϕ(t) is the random initial phase of light at time t and $E_{0}$ is the amplitude of the electromagnetic wave of light. Phase noise and nonlinear sweep noise are not considered to simplify the analysis. The sensing signal denoted by $E_{s,d}$ can be expressed as

(2) $$E_{s,d}(t)=E_{r}(t-\tau_{L})=E_{0}\exp\{j[2\pi f_{0}(t-\tau_{L})+\pi \gamma {\left(t-\tau_{L}\right)}^{2}+\phi_{v}]\},d=1,2$$

where $\phi_{v}$ is the phase change caused by external disturbances and $\tau_{L}=nL/c$ is the transit time of light through the sensing fiber. n, L, and c are the effective index of the fiber core, the length of the sensing fiber, and the speed of light in a vacuum, respectively. Then the interference signal $I_{d}$ of MZI can be expressed as

(3) $$I_{d}(t)={\left|E_{r,d}(t)+E_{s,d}(t)\right|}^{2}=E_{0}^{2}\{1+2\cos[2\pi (f_{0}\tau_{L}+f_{b}t+\frac{1}{2}\gamma \tau_{L}^{2})+\phi_{v}]\},d=1,2$$

where $f_{b}=\gamma \tau_{L}$ is the beat frequency between the sensing and the reference signals. Due to the forward-transmission frequency sweep, $f_{b}$ remains a constant and it is not affected by scattering points along the optical fiber.

The key step in locating vibrations along the sensing fiber in FTDVS structures is to calculate the time delay between two vibration-modulated phases received by the two BPDs. To simplify the phase-based mathematical model, the effects of optical attenuation and DC components are neglected.

Equation (3) can be simplified to

(4) $$\left\{\begin{array}{l} I_{1}(t)=\cos[2\pi f_{b}t+\phi_{v}(t-t_{1})]\\ I_{2}(t)=\cos[2\pi f_{b}t+\phi_{v}(t-t_{2})] \end{array}\right.$$

where $t_{1}=(L-Z)\times n/c$ and $t_{2}=Z\times n/c$ are the arrival times of the probe light after departing from the vibration position Z respectively. Assuming Δt is the time delay between the vibration-modulated phase signals at the detection ends of two MZIs, Δt can be expressed as

(5) $$\Delta t=t_{1}-t_{2}=\frac{(L-Z)\times n}{c}-\frac{Z\times n}{c}=\frac{n(L-2Z)}{c}$$

Then the vibration position Z can be obtained by the following equation:

(6) $$Z=\frac{L-\Delta t\times c}{2\times n}$$

It can be seen from the above equation that the vibration position is only related to the time delay difference Δt, which can be determined by applying the cross-correlation algorithm between the pair of counter-propagating phase modulation signals: $\phi_{1}(t)=\phi_{v}(t-t_{1})$ and $\phi_{2}(t)=\phi_{v}(t-t_{2})$. Due to the fact that two phase signals are generated by the same vibration source, they have a high degree of similarity. Hence, it is straightforward to obtain the time delay difference Δt between two phase signals using cross-correlation. The corresponding spatial resolution $SR_{FTDVS}$ can be expressed as

(7) $$SR_{FTDVS}=\frac{c}{n\times f_{s}}$$

where $f_{s}$ is the sampling rate. The phase demodulation module is implemented through digital down-conversion. The details are described in detail in the following section (take $I_{1}$ as an example). Given the frequency shift $f_{b}$, the in-phase carrier $I_{carrier\_i}$ in the IQ (in-phase, quadrature) demodulation algorithm [29] can be expressed as

(8) $$I_{carrier\_i}(t)=2\cos(2\pi f_{b}t)$$

The multiplication of $I_{1}$ and $I_{carrier\_i}$ yields

(9) $$I_{1}(t)\cdot I_{carrier{\_}_{i}}(t)=\cos[4\pi f_{b}t+\phi_{1}(t)]+\cos\phi_{1}(t).$$

Similarly, the out-of-phase carrier, $I_{carrier\_q}$ is defined as

(10) $$I_{carrier\_q}(t)=-2\sin(2\pi f_{b}t).$$

The corresponding multiplication product can be obtained:

(11) $$I_{1}(t)\cdot I_{carrier\_q}(t)=\cos[4\pi f_{b}t+\phi_{1}(t)]+\sin\phi_{1}(t).$$

High-frequency components can be filtered to obtain $i(t)=\cos\phi_{1}(t)$ and $q(t)=\sin\phi_{1}(t)$ through down-sampling. The phase demodulation module can extract the phase shift as follows:

(12) $$\phi_{1}(t)=\tan^{-1}\frac{q(t)}{i(t)}=\tan^{-1}\frac{\sin\phi_{1}(t)}{\cos\phi_{1}(t)}$$

If different photodetectors exhibit significantly different characteristics, the inconsistent amplitude of the light intensity at both ends would have little impact on the results, since it affects both the sensing light and the reference light, resulting in a similar phase response.

**Figure 2:**
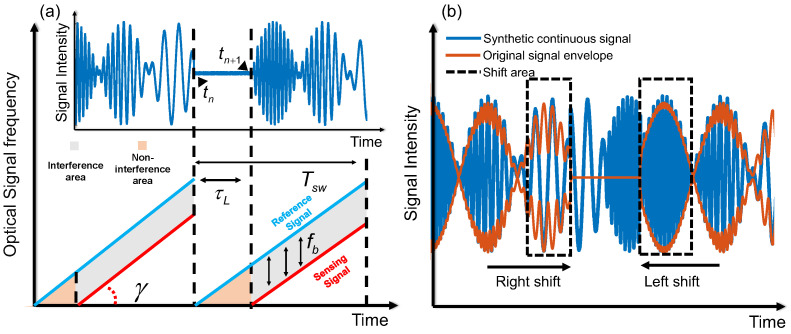
Working principles of the hybrid sensing system: (**a**) interference areas and non-interference areas; (**b**) illustration of the neighbor signal replacement method.

Note that the length of the reference path is much shorter than that of the sensing path. The BPD of the MZI detects only the reference signal at the beginning of a sweep period $T_{sw}$, which is approximately equal in length to $\tau_{L}$, as shown in Figure 2a. Signals received during the $\tau_{L}$ period belong to the non-interference area (NIA), and signals received within the period $T_{sw}-\tau_{L}$ belong to the interference area (IA). In coherent detection, no phase information is detected within NIA, leading to discontinuities in low-frequency phase demodulation. This is detrimental to the accurate recovery of vibration signals. To ensure the integrity of the demodulated signal as much as possible, a neighbor signal replacement (NSR) method is proposed. This method operates directly on interference intensity signals rather than phase signals. Compared to the approach of demodulating each IA segment followed by interpolation, it offers superior phase continuity and implementation simplicity by eliminating the need to adjust the initial phase of each IA segment. The schematic of NSR method can be shown at Figure 2b. Firstly, it is necessary to find the starting point $t_{n}$ and ending point $t_{n+1}$ of the NIA. Since the optical power in the IA is much greater than that of the NIA, it is straightforward to find the $t_{n}$ and $t_{n+1}$ of the NIA through a threshold peak-searching algorithm. Due to the interference effect, the difference in light intensity signals between the IA and the NIA is very large. Therefore, the average light intensity in the IA can be taken as the minimum peak point. In addition, the length of the non-interference area is fixed, and the starting and ending points can be further determined by determining the time interval between the previous minimum peak point and the next minimum peak point. Then the first half of the NIA is replaced with a part of equal length from the previous IA. Likewise, the second half of the NIA is replaced with a part of equal length from the next IA. The entire signal obtained using NSR is referred to as a synthetic continuous signal.

## 3. Experimental Results

### 3.1. Demodulation Performance Analysis of the NSR Method

The hybrid system folds the double-ended configuration such that the two detection ends are grouped at the same end, which solves the synchronization issue. The resulting fiber loop is suitable for applications involving a perimeter or allows a roundtrip. The voltage–strain coefficient of PTZ was measured to be 1.6606 με/V. $T_{sw}$ is 10 ms.

First, sinusoidal drive voltages with frequencies ranging from 300 Hz to 900 Hz were applied to the PZT and the detection system acquired an extended-duration optical interference intensity signal (lasting longer than multiple $T_{sw}$ periods) to validate the demodulation performance of the NSR method for different vibration frequencies. For comparison, the commonly used one-dimensional interpolation method was also applied to the dead-zone phase intervals. The specific procedure is as follows: demodulate each IA segment individually, then interpolate the NIA in the middle of each IA segment using Linear, Spline, and Pchip methods. Both zero-lag normalized cross-correlation (Zero-lag NCC) and Root Mean Square Error (RMSE) metrics were employed to assess the signal integrity and the vibration signal reconstruction accuracy of the NSR method versus the one-dimensional interpolation method. The expressions of zero-lag NCC are as follows:

(13) $$\rho_{\phi_{v},y}(0)=\frac{\sum \phi_{v}(n)\cdot y(t)}{\sqrt{\sum {\left\|\phi_{v}(t)\right\|}^{2}}\cdot \sqrt{\sum {\left\|y(n)\right\|}^{2}}}$$

where $\rho_{\phi_{v},y}(0)\in [-1,1]$ is the normalized correlation coefficient, which indicates the similarity between the demodulated phase signal $\phi_{v}$ and the simulated ground truth signal *y*. The amplitude, initial phase and frequency of *y* are set to be consistent with those of the demodulation phase. The expressions of RMSE are as follows:

(14) $$RMSE=\sqrt{\int {\left(\phi_{v}(n)-y(n)\right)}^{2}}$$

**Figure 3:**
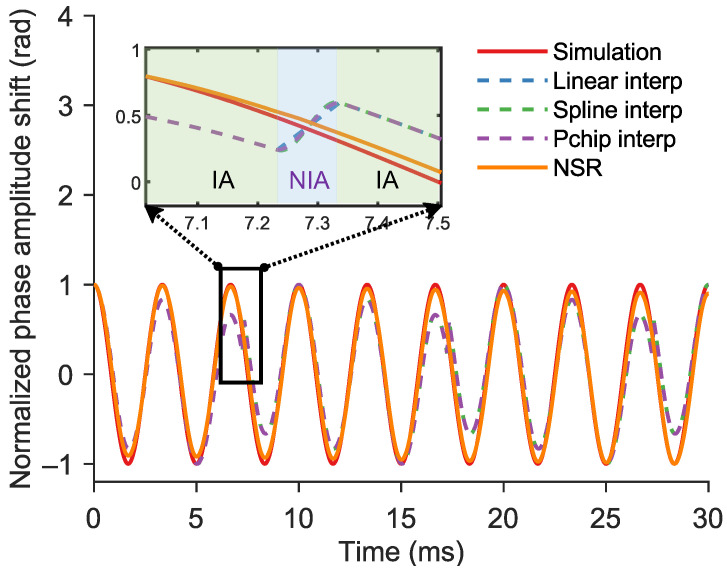
Comparison of 300 Hz sinusoidal phase signals reconstructed by the NSR method and three interpolation methods against the simulated ground truth signal.

The RMSE quantifies the magnitude of average error between demodulated phase signals $\phi_{v}$ and y. Under ideal conditions, the RMSE approaches zero. Figure 3 shows the phase signal curve of NSR method and the three interpolation methods. Figure 3 compares the 300 Hz sinusoidal phase signals reconstructed by the NSR method and three interpolation methods against the simulated ground truth signal. As observed in the figure, demodulating each IA segment individually causes the start and end points of the NIA segments to deviate from the original trend. This results in amplitude fluctuations across the full-phase signal after interpolation. In contrast, the NSR method directly applies time-shifting to the original time-domain signal, eliminating the need to adjust the initial phase of each IA segment. On the other hand, conventional 1D interpolation methods cannot be directly applied to interferometric intensity signals, as they struggle to accurately fit such broadband and complex waveforms. Therefore, phase demodulation must first be performed segment-wise before interpolation can be applied, which is a process that is more complex compared to the NSR approach. Consequently, the NSR method achieves better continuity compared to the demodulate-then-interpolate approach.

**Figure 4:**
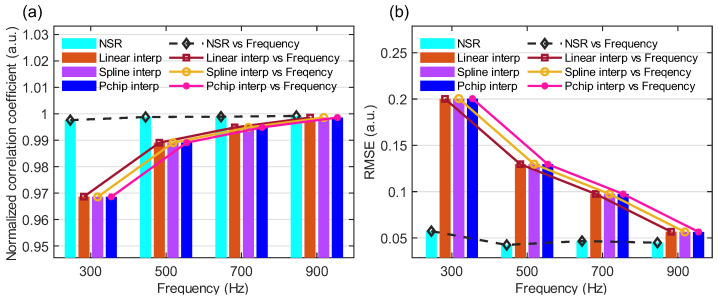
Performance comparison between the four reconstructed signals: (**a**) zero-lag NCC histogram of four methods; (**b**) RMSE histogram of the four methods.

Figure 4 compares the Zero-lag NCC and RMSE metrics of the NSR method against three interpolation methods for sinusoidal vibration frequencies ranging from 300 Hz to 900 Hz. As shown in Figure 4a, while the $R_{\phi_{v}y}(0)$ of all four methods increases with increasing frequency, the NSR method maintains a significantly higher $R_{\phi_{v}y}(0)$ (0.9976) at 300 Hz compared to the interpolation methods.

The $\rho_{\phi_{v},y}(0)$ can be converted into signal-to-noise ratio using the following equation:

(15) $$SNR=\frac{{\rho^{2}}_{\phi ,y}(0)}{1-{\rho^{2}}_{\phi ,y}(0)}$$

The derivation of this equation is provided in Appendix A.1. For the NSR method, the normalized zero-lag value at 300 Hz is 0.9976, which corresponds to an SNR of 54.37 dB. This indicates that the NSR method provides an excellent SNR.

As shown in the Figure 4b, similarly, the RMSE of all methods decreases with increasing frequency, yet the NSR method achieves the lowest RMSE (0.057) at 300 Hz among all techniques in the low-frequency range. These results demonstrate that the NSR method effectively reconstructs signals in the NIA even for low-frequency vibrations, outperforming one-dimensional interpolation approaches.

The RMSE can be converted into signal-to-noise ratio using the following equation:

(16) $$SNR_{\log}=10\log_{10}\frac{P_{y}}{RMSE^{2}}$$

where $P_{y}$ is the power of y(t). For the NSR method at 300 Hz, the RMSE value is 0.057 and $P_{y}=11,719$, which corresponds to a calculated SNR of 75.57 dB. Thus, characterization using the RMSE yields an even better SNR.

### 3.2. FTDVS Module: Long Distance and Dynamic Measurements

**Figure 5:**
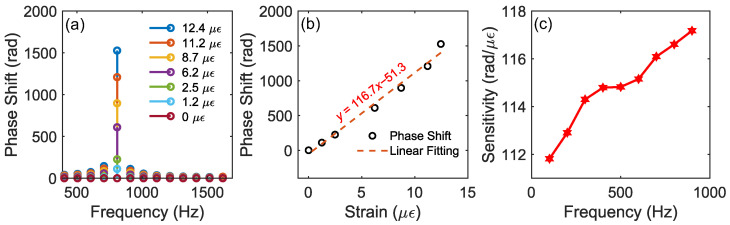
Sensitivity and its stability: (**a**) frequency spectrum of demodulated phase signal under different strains with 800 Hz sinewave; (**b**) relationship between strain and phase shift; (**c**) frequency-dependent sensitivity curve at a constant PZT drive amplitude of 15 V.

For the FTDVS module, by characterizing the phase shift under different fiber strains, their linear relationship, and thus the sensitivity, was explored. When an external vibration occurs at a certain position of the sensing fiber link, the refractive index of the fiber core and the fiber birefringence will change accordingly, which in turn will induce a phase delay in the transmission light. There is a linear relationship between vibration-induced strain and phase shift. The amplitude and frequency can be extracted through a fast Fourier transform (FFT) on the phase signal. As shown in Figure 5a, by applying an 800 Hz sinusoidal signal with different drive voltages (from 0 V to 15 V) to the PZT, the corresponding phase shifts were measured. A linear fit between strain and phase shift is plotted in Figure 5b. The gradient (or sensitivity) of strain vs. phase shift obtained from the linear fit is 116.71 rad/με. In order to test the repeatability of the response of the FTDVS module at different frequencies, sinusoidal drive signals with a fixed voltage at 15 V from 100 Hz to 900 Hz were applied to PZT. Figure 5c shows the frequency-dependent sensitivity curve, where the results show that under different vibration frequencies, the sensitivity remains relatively stable from 111.8 to 117.1 rad/με. The limit of detection (LoD) of the FTDVS module can be calculated by the following equation:

(17) $$LoD=\frac{3.3\times std(noise)}{sensitivity\times \sqrt{B}}$$

where $noise=\phi_{noise}(t)$ is the noise-equivalent phase shift in the absence of vibration, $\sqrt{B}$ is the square root of bandwidth (400 MHz). Therefore, the LoD of FTDVS module vibration is 2.3 $p\varepsilon /\sqrt{Hz}$.

**Figure 6:**
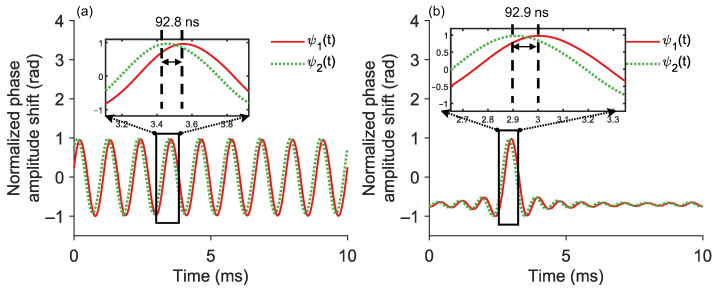
Time-domain signals for vibration positioning: (**a**) demodulated phase signal, 900 Hz sinusoidal; (**b**) demodulated phase signal, 100 Hz sinc.

Since FTDVS usually uses a fixed-wavelength laser source, the change to a swept wavelength design requires the positioning and demodulation ability of the FTDVS module to be verified. Through down-conversion, the beat signal frequency is shifted to the baseband and second harmonic, while retaining the I/Q components. Then a 1/10 down-sampling ratio is used to filter out the second harmonic of the I/Q components. Figure 6 shows the MZI data acquired from BPD4 and BPD3 when vibration was applied around 20 km. Figure 6a,b shows the demodulated phase signals $\phi_{1}(t)$ and $\phi_{2}(t)$ when the PZT induces a 900 Hz sinusoidal signal and 100 Hz sinc signal, respectively. It is clear that the method of down-conversion and down-sampling is feasible for restoring high-frequency vibration information. The delay between the two phase signals resulting from a 900 Hz sinusoidal PZT signal is 92.8 ns, while the delay associated with a 100 Hz sinc PZT signal is 92.9 ns. The corresponding vibration positions deduced according to Equation (5) are 20,065 m and 20,052 m, respectively.

**Figure 7:**
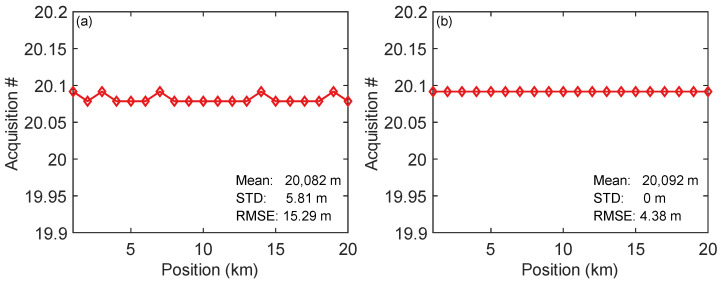
Vibration positioning repeatability for a 21,127 m length fiber link: (**a**) 900 Hz sinusoidal signal; (**b**) 100 Hz sinc signal. STD: standard deviation; RMSE: root mean squared error.

For characterizing the repeatability of vibration positioning, repetitive vibration measurements were carried out. In the experiment, 20 sets of 900 Hz sinusoidal vibrations and 100 Hz sinc vibrations were collected separately. Each measurement duration is 500 ms, ensuring that sufficient cycles of the signal are present for accurate analysis. The results of the experiments are shown in Figure 7. According to the figure, for the 900 Hz single-frequency vibration, the average position is 20,082 m with an STD of 5.81 m, and the RMSE relative to the true position is 15.29 m. For the 100 Hz multi-frequency sinc vibration, the average position is 20,092 m with an STD of 0 m, and the RMSE relative to the true position is 4.38 m. This demonstrates that the NSR method successfully mitigates NIA-induced demodulation discontinuities, enabling precise position of both single-frequency (900 Hz sinusoidal) and broadband (100 Hz sinc) vibrations with RMSEs of 15.29 m and 4.38 m, respectively.

### 3.3. OFDR Module: Low-Frequency Measurements

**Figure 8:**
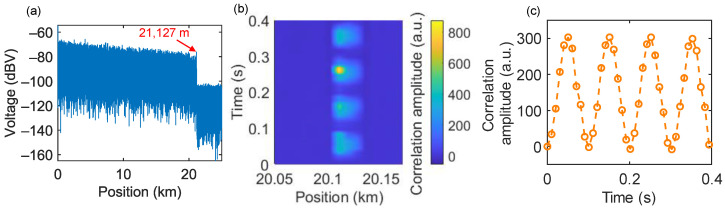
OFDR sensing results: (**a**) Rayleigh backscatter signal as a function of fiber length with a sweep range of 1 GHz; (**b**) time–distance–correlation relationship for a 10 Hz sinusoidal signal at a position of 20,118 m; (**c**) time-dependent cross-correlation amplitude.

Due to the general reliance on the cross-correlation algorithm and AC-coupled phase extraction algorithms such as IQ and differential cross-multiplication (DCM), FTDVS suffers from limitations in detecting low-frequency vibrations and static strain. Therefore, integration of an OFDR module into a modified FTDVS (swept wavelength) can compensate for the missing functionality and enable detection of low-frequency vibration and static strain. The fast Fourier transform (FFT) of the RBS signal received by BPD1 and BPD2 converts the optical frequency-domain signal into the distance domain, as shown in Figure 8a. It can be seen that the RBS experiences significant attenuation at 21,127 m, which is exactly equal to the length of the sensing fiber. The SR of the OFDR is expressed by the following equation:

(18) $$SR_{OFDR}=\frac{cN}{2nF}$$

where *N* is the length of cross-correlation window, F = 1 GHz is the bandwidth, and the corresponding spatial resolution is 0.1022 m.

To test low-frequency vibrations, a sinusoidal drive signal with a frequency of 10 Hz and a Vpp of 0.45 V was applied to PZT as the vibration source. BPD2 collected two sets of data as the measurement signal and reference signal. Both sets of received signals were subjected to FFT to obtain the power spectrum of reflected light at different scattering points, then the optical frequency domain was converted into the distance domain, after being divided into fiber segments with a sliding window width of 80 points. Subsequently, the inverse Fourier transform (IFFT) was performed on each of these fiber segments to transform them back into the optical frequency domain. Through the IFFT data processing, a Rayleigh backscattering spectrum as a function of wavelength shift was obtained. Finally, cross-correlation was carried out on each fiber segment between the reference and measured signals to determine their wavelength shift within a localized range of beat frequencies. When an external perturbation is applied, the signal of each fiber segment experiences a shift, and the position of the perturbation can be obtained through the cross-correlation algorithm. Repeating the above operations and analyzing the time-varying change in each fiber segment (i.e., slow-time axis) can retrieve the vibration signal at a specific position along the fiber, as shown in Figure 8b. According to Equation (14), the SR is 8.17 m. Note that to increase the number of cross-correlation points, zero padding was performed on each fiber segment data before IFFT. By extracting the time-domain signal at 20,118 m, the wavelength shift results with a frequency of 10 Hz is shown in Figure 8c.

### 3.4. Frequency-Dependent Positioning Performance

When the measured SNR is high, the positioning error is mainly induced by delay value fluctuation of the peak of the cross-correlation function, and MSE will follow the Crammer–Rao lower bound (CRLB) [30]:

(19) $$MSE_{CRLB}=\frac{3}{8\pi^{2}}\cdot \frac{1+2SNR}{SNR^{2}}\cdot \frac{1}{{B_{S}}^{3}T}$$

where *T* is the observation time and *B_S_* is the bandwidth of vibration signal.

Consequently, under identical measurement durations, the vibration position based on cross-correlation yields reduced accuracy. Under such conditions, the single-mode FTDVS system fails to sustain a reliable positioning performance. By incorporating the OFDR module, the hybrid system maintains high-precision position capabilities at low frequencies (<$1/2T_{sw}$). An experimental validation was performed by applying a 10–900 Hz sinusoidal signal to a PZT. For the position accuracy at each frequency, the RMSE between the calculated positions and the true vibration position were calculated from four repeated trials in the FTDVS module. In the hybrid system under low-frequency conditions (<$1/2T_{sw}$), the position value was determined by identifying the position corresponding to the peak wavelength shift between the reference and measurement signals within each fiber segment of the OFDR data. Similarly, the RMSE of positions were computed from four repeated trials.

**Figure 9:**
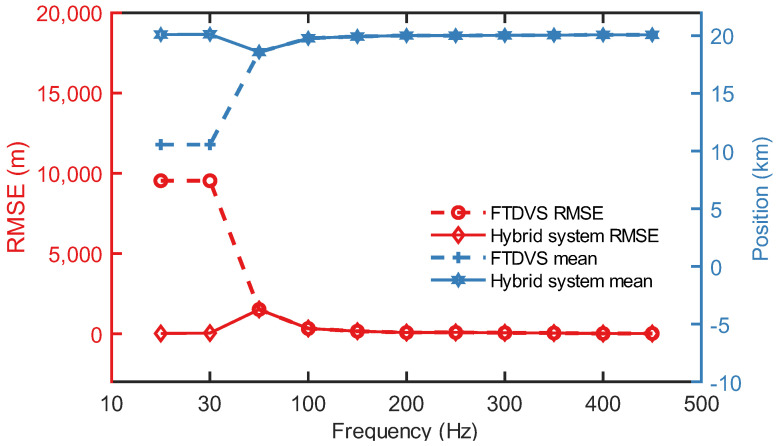
Comparison of positioning ability for the single-mode FTDVS and the hybrid system.

The experimental results are shown in Figure 9. For the single-mode FTDVS system, the positioning errors for 10 Hz and 30 Hz single-frequency vibrations are significant. The RMSE for both is 9352 m, and the average position across four repeated trials is 10.564 m. The corresponding time delay calculated via cross-correlation is 0 s. This indicates that at low frequencies, the single-mode FTDVS system loses its capability for accurate vibration positioning. In contrast, the hybrid system provides high-precision position ability in the low-frequency range. The RMSE values for 10 Hz and 50 Hz are 13.1 m and 37.4 m, respectively. This level of accuracy approaches the position accuracy achieved by the FTDVS module with high-frequency signals. Furthermore, the mean values from the four repeated trials are 20,109 m and 20,129 m, differing from the actual vibration position by only 13 m and 33 m, respectively. This clearly demonstrates that the hybrid system can effectively address the inaccurate low-frequency vibration positioning problem inherent in cross-correlation-based FTDVS systems.

### 3.5. Long-Range Position Capability of the Hybrid System

**Figure 10:**
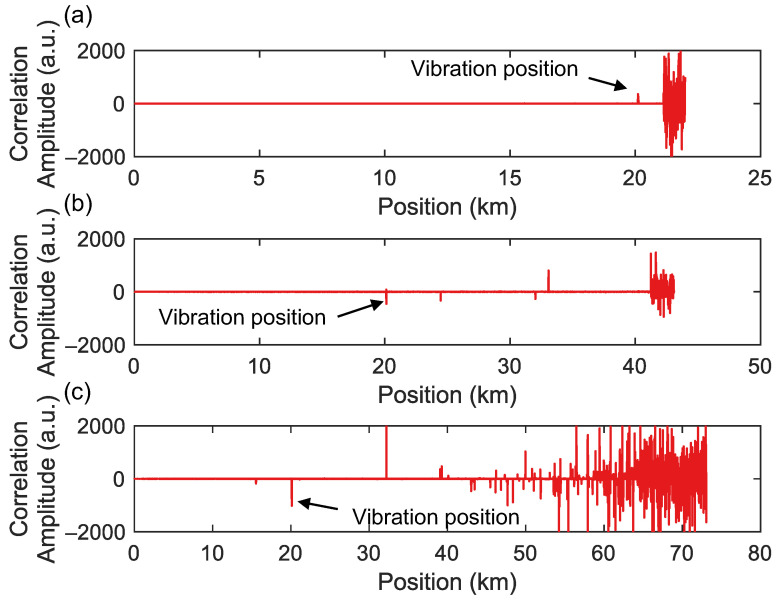
Cross-correlation results in OFDR for different fiber lengths: (**a**) 21,127 m; (**b**) 41,240 m; and (**c**) 71,710 m.

**Figure 11:**
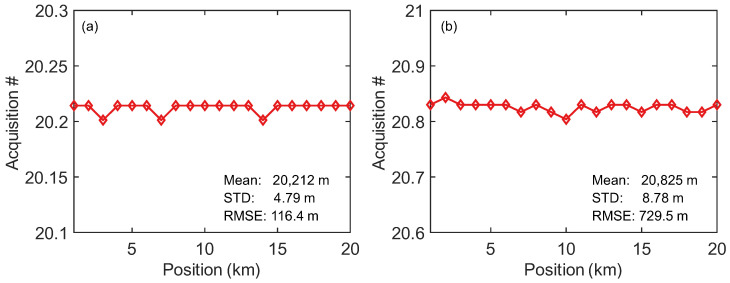
Long-distance vibration positioning results with 800 Hz sine signal: (**a**) 41,240 m; (**b**) 71,710 m sensing fiber length.

To study the system performance over longer distances, the sensing fiber length was increased to 41,240 m and 71,710 m, with the PZT position maintained at 20,096 m. Considering the significant attenuation of light over such long distances, an EDFA was used in front of OC1 to amplify the light intensity. A PZT drive signal with a frequency of 10 Hz and a Vpp of 0.45 V was used as a vibration source. Figure 10 shows the cross-correlation results from the OFDR when probing sensing fiber with different lengths. It is evident that as the sensing fiber length increases, the positioning accuracy of the OFDR decreases significantly. Especially when the sensing fiber length is 71,710 m, there is significant noise in the latter half of the sensing distance. For longer-range OFDR systems, since the ratio of Rayleigh backscattering to forward-propagating light is −57 dB, the Rayleigh backscattered signal at the distal end decreases significantly as the length of the sensing fiber increases. Secondly, in long-distance sensing, the phase noise inherent to the light source itself increases with the length difference between the sensing arm and the reference arm. As a result, the phase noise at the end of the OFDR sensing fiber also becomes more pronounced. Employing bidirectional amplification techniques such as Raman amplification can enhance the optical power of the sensing signal, thereby extending the sensing range. Another approach is using an auxiliary interferometer to estimate the phase noise of the light source, which can then be compensated in the OFDR sensing signal to reduce phase noise at the end of the fiber. The sensing distance of conventional OFDRs is usually at the 10–100 m scale, but the long-range variant in this work operates up to 41 km. Correspondingly, when a drive signal with a frequency of 800 Hz and VPP of 15 V is applied to PZT, the repeated positioning results of FTDVS are shown in Figure 11. The positioning accuracies (STD) for sensing fiber lengths of 41,420 m and 71,710 m are 4.69 m and 8.52 m, respectively. These distance errors are not significantly different from those of the sensing fiber length of 21,127 m. This means that for longer-distance sensing, FTDVS can provide reliable vibration positioning, while OFDR experiences difficulty.

## 4. Conclusions

Although FTDVS has the advantage of a longer single-span sensing distance, due to the typical reliance on the cross-correlation algorithm and AC-coupled phase extraction algorithms such as IQ and DCM, it has limitations in detecting low-frequency vibrations and static strain. To address these problems, this paper proposed a hybrid distributed fiber-optic sensing technology based on a combination of FTDVS and OFDR. For the probe light, the hybrid system uses stable linear optical frequency sweeping facilitated by high-order sideband injection locking, and integrates the OFDR module into a modified FTDVS (which shares a swept wavelength laser source), which can compensate for the missing functionality and enable detection of low-frequency vibration and static strain.

Although the hybrid system combines two technologies together, it is far from a simple integration. OFDR uses swept wavelength (backscattered light) while conventional forward transmission distributed vibration sensing (FTDVS) uses fixed wavelength (forward-transmitted light). Hence, the demodulation algorithms of the latter need to be modified in order to share the same laser source as OFDR. To the best of our knowledge, this is the first reported instance of combining FTDVS and OFDR into a unified system with a shared laser source. In sensing systems, the light source and deployment of sensing fiber account for a significant portion of the total cost. Therefore, sharing the laser source and sensing fibers between the two sensing modules in the hybrid system can considerably reduce costs and improve the reusability of system components. Table 1 lists the cost proportions of key components in the system and indicates whether or not they are shared in the hybrid system. In terms of performance, though individual specifications of the hybrid system cannot surpass that of a standalone system (FTDVS or OFDR), the hybrid system possess a more comprehensive range of functions that is unattainable with a standalone system. For example, the OFDR module can to some extent compensate for the difficulty in achieving static strain measurement and multi-point vibration positioning in FTDVS systems. Correspondingly, the FTDVS module can compensate for the weak signal-to-noise ratio of backscattered light after long-distance transmission (otherwise limited range) and achieve a wider frequency response (limited by the swept period). Hence, the hybrid system can realize both short- and long-distance sensing, in addition to static and dynamic strain analysis.

To summarize, the characteristics of a new hybrid distributed sensing architecture were studied, combining FTDVS and OFDR. The experimental results demonstrate that the hybrid system can locate vibration signals along the sensing fiber with frequencies ranging from 0 to 900 Hz (OFDR inherently can measure static strain [28]) and a sensing distance up to 21 km. The FTDVS module demonstrated a positioning accuracy of 5.81 m (for precisely locating intrusion attempts such as cutting or climbing fences), an LoD of 2.3 $p\varepsilon /\sqrt{Hz}$, and vibration frequency range of 0–900 Hz (for analyzing intrusion types based on vibration signatures, such as digging, approaching vehicles, or animal contact). Even with a sensing distance of 71 km, the hybrid system can still perform positioning of high-frequency vibration signals via the FTDVS module, which can reduce the number of units needed and lower the operating cost. The all-round hybrid sensing system offers significant benefits for real-time, wide-coverage monitoring of critical infrastructure perimeters, including airports and power plants. No data averaging was performed during signal processing to accelerate the real-time processing speed. It was found that the use of linear optical frequency sweeping did not affect the positioning ability of FTDVS.

## Figures and Tables

**Table 1:** The cost of key components in the system.

	Material Cost	Labor Cost	Shared or Not
Laser source	High	Medium	Yes
Photodetectors	Medium	Low	No
Sensing fibers	Medium	High	Yes
Optical circulators	Low	Low	No
Optical couplers	Low	Low	No
Oscilloscope	Medium	Medium	Yes

## Data Availability

The data underlying the results presented in this paper can be made available upon request.

## Appendix A

### Appendix A.1. The Process of Deriving Signal-to-Noise Ratio from Zero-Lag NCC

First, we assume that the laser phase noise and the environmental disturbance noise are additive to the phase change induced by the vibration. We also assume that both the observed/vibrational phase signal $\phi_{v}(t)$ and the noise n(t) have a zero mean (a condition satisfied by choosing a sinusoidal signal as the vibration source):

(A1) $$\phi_{v}(t)=y(t)+n(t)$$

Substitute it into the Zero-lag cross-correlation formula between the observed signal and the original signal:

(A2) $$R_{\phi_{v},y}(0)=E[\phi_{v}(t)y(t)]=E[(y(t)+n(t))y(t)]$$

where *E*[·] is the mathematical expectation. Since the original signal and noise are independent of each other,

(A3) $$R_{\phi_{v},y}(0)=E[y^{2}(t)]+E[y(t)n(t)]=R_{y,y}(0)+0=P_{y}$$

where $P_{y}=R_{y,y}(0)$ is the power of y(t). The autocorrelation of the observed signal can be calculated using the following formula:

(A4) $$R_{\phi_{v},\phi_{v}}(0)=E[{\phi_{v}}^{2}(t)]=E[{\left(y(t)+n(t)\right)}^{2}]=E[y^{2}(t)]+E[n^{2}(t)]=P_{y}+P_{n}$$

where $P_{n}=R_{n,n}(0)$ is the power of n(t). Substitute it into the normalized cross-correlation formula:

(A5) $$\rho_{\phi_{v},y}(0)=\frac{R_{\phi ,y}(0)}{\sqrt{R_{\phi_{v},\phi_{v}}(0)\cdot R_{y,y}(0)}}=\frac{P_{y}}{\sqrt{P_{y}\cdot (P_{y}+P_{n})}}$$

The SNR of a vibration signal is defined as

(A6) $$SNR=\frac{P_{y}}{P_{n}}$$

Substitute $P_{n}=P_{y}/SNR$ into $\rho_{\phi_{v},y}(0)$

(A7) $$\rho_{\phi_{v},y}(0)=\frac{P_{y}}{\sqrt{P_{y}\cdot (P_{y}+\frac{P_{y}}{SNR})}}=\sqrt{\frac{SNR}{SNR+1}}$$

Now we get the relationship between $\rho_{\phi_{v},y}(0)$ and SNR:

(A8) $$SNR=\frac{{\rho^{2}}_{\phi ,y}(0)}{1-{\rho^{2}}_{\phi ,y}(0)}$$

By substituting with commonly used logarithmic relationships, we can obtain

(A9) $$SNR_{\log}=10\log_{10}\frac{{\rho^{2}}_{\phi ,y}(0)}{1-{\rho^{2}}_{\phi ,y}(0)}$$

### Appendix A.2. The Process of Deriving the Signal-to-Noise Ratio from RMSE

For RMSE, according to its definition:

(A10) $$RMSE=\sqrt{\int \left(\phi_{v}(t)-y(t\right){)}^{2}}=\sqrt{\int n^{2}(t)}=\sqrt{P_{n}}$$

Similarly, substitute $P_{n}=P_{y}/SNR$ into RMSE and substitute it with commonly used logarithmic relationships:

(A11) $$RMSE=\sqrt{\frac{P_{y}}{SNR}}$$

Now we get the relationship between RMSE and SNR:

(A12) $$SNR_{\log}=10\log_{10}\frac{P_{y}}{RMSE^{2}}$$
